# Microglial-derived miRNA let-7 and HMGB1 contribute to ethanol-induced neurotoxicity via TLR7

**DOI:** 10.1186/s12974-017-0799-4

**Published:** 2017-01-25

**Authors:** Leon G. Coleman, Jian Zou, Fulton T. Crews

**Affiliations:** 1 Bowles Center for Alcohol Studies, The University of North Carolina School of Medicine, 104 Manning Drive, 1007 Thurston-Bowles Building, CB# 7178 UNC-CH, Chapel Hill, NC 27599 USA; 2 Bowles Center for Alcohol Studies, University of North Carolina at Chapel Hill, 104 Manning Drive, CB#7178, Thurston-Bowles Building Room 1007, Chapel Hill, NC 27599 USA

**Keywords:** Alcohol, Neurodegeneration, Toll-like receptor 7, Microvesicles, Amphoterin, Micro-RNA

## Abstract

**Background:**

Toll-like receptor (TLR) signaling is emerging as an important component of neurodegeneration. TLR7 senses viral RNA and certain endogenous miRNAs to initiate innate immune responses leading to neurodegeneration. Alcoholism is associated with hippocampal degeneration, with preclinical studies linking ethanol-induced neurodegeneration with central innate immune induction and TLR activation. The endogenous miRNA let-7b binds TLR7 to cause neurodegeneration.

**Methods:**

TLR7 and other immune markers were assessed in postmortem human hippocampal tissue that was obtained from the New South Wales Tissue Bank. Rat hippocampal-entorhinal cortex (HEC) slice culture was used to assess specific effects of ethanol on TLR7, let-7b, and microvesicles.

**Results:**

We report here that hippocampal tissue from postmortem human alcoholic brains shows increased expression of TLR7 and increased microglial activation. Using HEC slice culture, we found that ethanol induces TLR7 and let-7b expression. Ethanol caused TLR7-associated neuroimmune gene induction and initiated the release let-7b in microvesicles (MVs), enhancing TLR7-mediated neurotoxicity. Further, ethanol increased let-7b binding to the danger signaling molecule high mobility group box-1 (HMGB1) in MVs, while reducing let-7 binding to classical chaperone protein argonaute (Ago2). Flow cytometric analysis of MVs from HEC media and analysis of MVs from brain cell culture lines found that microglia were the primary source of let-7b and HMGB1-containing MVs.

**Conclusions:**

Our results identify that ethanol induces neuroimmune pathology involving the release of let-7b/HMGB1 complexes in microglia-derived microvesicles. This contributes to hippocampal neurodegeneration and may play a role in the pathology of alcoholism.

**Electronic supplementary material:**

The online version of this article (doi:10.1186/s12974-017-0799-4) contains supplementary material, which is available to authorized users.

## Background

The role of Toll-like receptors (TLRs) in innate immunity has recently been illuminated. TLRs recognize damage-associated molecular pattern molecules (DAMPs) to initiate innate immune signaling cascades. In the CNS, however, TLRs function not only as agents of immunity, but regulate neuronal morphology, pain, and neurodegeneration through recognition of endogenous agonists [1–3]. TLR7 is an endosomal TLR that recognizes endogenous micro-RNAs (miRs), single-stranded RNAs (ss), and short interfering (si) RNAs [4]. TLR7 signaling can lead to activation of transcription factors IRF7 or NFκB and cause either neuroimmune responses or neurodegeneration [4–7]. The endogenous TLR7 agonist miR let-7 has been found to cause neurodegeneration [6]. let-7b in particular has a GU-rich region that is readily recognized by TLR7. Studies utilizing miRNA profiling find increased expression of several let-7 isoforms in human and rodent brain after chronic alcohol [8, 9].

Both neuroimmune activation and neurodegeneration are inseparable in many CNS disorders including alcoholism [10], Parkinson’s [11, 12], and Alzheimer’s disease [13, 14]. Alcoholism is associated with progressive neurodegeneration throughout the brain including the hippocampus and cortex [15–17]. Neuroimmune activation precedes and exacerbates neurodegeneration [18–20]. Ethanol activates the neuroimmune system through TLR activation and the release of endogenous DAMPs, such as the TLR4 agonist high mobility group box-1 (HMGB1). Studies of postmortem human alcoholic brains, in vivo rodent studies, and in vitro hippocampal-entorhinal brain slice culture (HEC) show that ethanol increases the cortical expression of TLRs 2-4, HMGB1 [10, 21, 22]. Peripherally, ethanol causes the release of microvesicles (MVs) into the circulation that contain pro-inflammatory miRNAs [23, 24]. Since TLR7 activation causes both innate immune activation and neurodegeneration, we hypothesized that ethanol would activate TLR7 signaling leading to innate immune gene induction. We further hypothesized that ethanol would cause the release of the endogenous TLR7 agonist miR let-7b in MVs. HMGB1 is a nucleic acid binding protein that is released from neurons by ethanol [21, 22]. HMGB1 is required for immune responses to TLR7 agonists [25] and acts as a chaperone for DNA or cytokines potentiating their function through their native receptors [26–28]. HMGB1 is released in MVs and ethanol increases expression and active secretion of HMGB1 from the brain [22, 29]. Thus, we hypothesized that HMGB1 would bind miR let-7b in MVs to help facilitate its activity as an endogenous agonist of TLR7.

We report here in postmortem human alcoholic hippocampal brain tissue that TLR7, HMGB1, and the microglia activation marker CD11b are increased. Interestingly, TLR7 expression in human hippocampus correlated with lifetime alcohol intake, suggesting a role in the pathology of the disease. In rat HEC brain slice culture, we found that ethanol increases TLR7, HMGB1, IL-1β, TNFα, and let-7b consistent with findings in human alcoholics. Concomitant with the increase in TLR7, ethanol also increases the release of let-7b and HMGB1 in MVs and potentiates let-7b induced neurodegeneration via TLR7 activation. Using RNA immuno-purification followed by RT-PCR (RIP assay), we found that HMGB1 forms heterodimeric complexes with let-7b in MVs in response to ethanol. Using flow cytometry for MVs from HEC slice culture and analysis of SH-SY5Y neuronal and BV2 microglia cell lines, we found that the majority of microvesicular HMGB1 and let-7b are derived from microglia. Thus, we report here the identification of a novel inter-cellular communication mechanism in the pathology of alcohol abuse, whereby ethanol causes the release of HMGB1-let-7 complexes in MV from microglia.

## Methods

### Reagents

The following reagents were purchased from Sigma-Aldrich (St Louis, USA): imiquimod (I5159) and glycyrrhizin (G2137). HMGB1 ELISA kit was purchased from IBL International (Hamburg, Germany); primary antibodies from Novus Biologicals: Na^+^/K^+^ ATPase α3 (NB300-540APC), GFAP (NBP2-34401 V2), CD11b (NBP2-34678PE), and HMGB1 (NB100-2322AF488); and primary antibodies from Abcam: CD11b for western blot (ab75476), argonaute 2 (ab32381).

### Hippocampal-entorhinal cortex slice culture

All protocols followed in this study were approved by the Institutional Animal Care Use Committee at UNC and were in accordance with National Institute of Health regulation for the care and use of animal in research. Organotypic brain slice cultures are prepared as described previously [22]. Briefly, the hippocampal entorhinal region is dissected and sliced transversely from post-natal day 7 rat pups. Slices are 375 μm thick. HEC slices were placed onto tissue insert membrane (10 slices/insert) and cultured with medium containing 75% MEM with 25 mM HEPES and Hank’s salts, 25% horse serum (HS), 5.5 g/L glucose, 2 mM L-glutamine in a humidified 5% CO_2_ incubator at 36.5 °C for 7 days in vitro (DIV), followed by 4 DIV in medium containing 12.5% HS and then 3 DIV in serum-free medium supplemented with N2. The cultures after 14 DIV were used for experiments and drug treatments with serum-free N2-supplemented medium. For ethanol exposures, slices were exposed to ethanol (25–100 mM) for 48 h. For let-7b mimic exposure studies, slices were treated with DOTAP, DOTAP plus let-7b mimic, or let-7b mimic plus ethanol for 48 h. For TLR7 agonist enhancement studies, slices were treated with either 500 ng/mL of imiquimod (IMQ) or vehicle for 48 h, followed by addition of either ethanol (100 mM) or vehicle for 96 h.

### Immunofluorescent staining and analysis

HEC slice cultures were removed at the end of the experiment and fixed with 4% paraformaldehyde with 5% sucrose in 0.01 M PBS for 24 h at 4 °C. All primary antibodies were incubated for 48 h at 4 °C. Either Alexa Fluor 594 or Alexa Fluor 488 secondary antibodies (1:2000; Molecular Probes, Eugene, OR) were used for immunofluorescent staining and incubated for 1 h at room temperature. The slices were coverslipped with anti-fade mounting medium (pro-long; Molecular Probes). Images were obtained using a LeicaSP2 AOBS Upright Laser Scanning Confocal in Michael Hooker Microscopy Facility (University of North Carolina, Chapel Hill, NC). Fluorescent pixel density analysis was performed using ImageJ^TM^ software.

### Neuronal and microglial cell line experiments

SH-SY5Y and BV2 cells were allowed to grow in culture as described previously in standard cell culture conditions [30, 31]. For BV2 microglia and SH-SY5Y neuronal experiments, 3 × 10^5^ cells/well were plated on 6-well Corning^TM^ cell culture plates. Cells were allowed to adhere overnight, and the next morning, they were treated with ethanol (100 mM) for 24 h.

### Postmortem human alcoholic analyses

Frozen postmortem human hippocampal tissue was obtained from the New South Wales Brain Tissue Bank in Sidney Australia as described previously [21]. Approximately 100 mg of tissue was provided for each subject. Given the small fragments of tissue, morphological assessment was not possible. Individuals with comorbid liver cirrhosis or nutritional deficiencies were excluded. The leading common cause of death was cardiovascular disease for both groups (16/18). Postmortem intervals were also documented and did not correlate with mRNA measurements. Psychiatric and alcohol use disorder diagnoses were confirmed using the Diagnostic Instrument for Brain Studies, which is in compliance with the Diagnostic Statistical Manual of Mental Disorders [32]. Details for each subject are provided in Table 2.

### Isolation of mRNA, miRNA, and quantification via RT-PCR

Isolation of mRNA was performed as previously [22]. Briefly, total RNA or miRNA were isolated using RNeasy Mini Kit or the miRNeasy Mini Kit respectively (QIAGEN Inc., CA). RNA quantification was performed using a nanodrop 2000^TM^ spectrophotometer. RIN scores were obtained after RNA isolation using the Agilent Bioanalyzer 2100 (Agilent Technologies, Santa Clara, CA) to determine RNA integrity as described previously [33]. For mRNA reverse transcription, 2 μg of RNA was used to synthesize cDNA using random primers (Invitrogen) and reverse transcriptase Moloney murine leukemia virus (Invitrogen). The primer sequences used for reverse transcriptase of mRNA targets are included in Table 1. Genes of interest were normalized to housekeeping genes β actin for mRNA analyses and snRU6 for miRNA analyses. Importantly, ethanol did not cause significant reductions in β actin, indicating its adequacy as a housekeeping gene. For miRNA, TaqMan^TM^ Advanced miRNA cDNA Synthesis Kit was used for reverse transcriptase. The known sequences for the measured miRNAs were mature miR let-7b (UGAGGUAGUAGGUUGUGUGGUU), miR-155 (CUCCUACCUGUUAGCAUUAAC), and miR 181c AACAUUCAACCUGUCGGUGAGU.

**Table 1:** Primers used for mRNA and miRNA quantification by RT-PCR

Target	Forward (5′ to 3′)	Reverse (5′ to 3′)
IL-1β	GAAACAGCAATGGTCGGGAC	AAGACACGGGTTCCATGGTG
TNFα	AGCCCTGGTATGAGCCCATGTA	CCGGACTCCGTGATGTCTAAG
TLR7	AGCTCTGTTCTCCTCCACCA	CATGGGTGTTTGTGCTATCG
β-actin	CTACAATGAGCTGCGTGTGGC	CAGGTCCAGACGCAGGATGGC

### Microvesicle isolation

MVs are isolated by sequential centrifugation as described previously [29]. Briefly, media was centrifuged at 2000*g* for 20 min to remove cells. Supernatant was then centrifuged at 10,000*g* for 30 min to remove cellular debris. Remaining supernatant was then centrifuged at 21,000*g* for 1 h. The MV-containing pellet was washed in PBS and centrifuged again at 21,000*g*. The MV pellet was suspended in the appropriate buffer for analysis. This preparation results in MVs ranging between 100 nm and 1 μm in diameter.

### ELISA measurements of HMGB1

Media HMGB1 levels were determined from undiluted media by ELISA (IBL, Germany) according to the manufacturer’s instruction. Tissue levels of HMGB1 from human hippocampus were measured by ELISA. Samples were first treated with perchloric acid (BioVision catalog #K808) to separate HMGB1 from its binding partners as previously described [34]. Purified supernatant was then assessed by ELISA at a dilution of 1:25.

### Flow cytometric analysis of microvesicle cellular origin

Composition and cell origin of MVs was done as described [35, 36]. Briefly, MVs were permeabilized with Fix/Perm buffer (Biolegend), incubated with Fc blocking buffer (Biolegend), and labeled using antibodies to HMGB1, GFAP (astrocytes), Na^+^/K^+^ ATPase α3 (neurons), and CD11b (microglia). Samples are incubated with fluorescent secondary antibodies when appropriate. The Stratedigm S1000Ex was used to assess the stained MVs at the UNC Flow Cytometry Core Facility. Size gates to identify MVs (0.1–1.0 μm) were set using MegaMix^TM^ (BioCytex) size gating beads (Additional file 1: Figure S1A). Single color controls for each primary antibody, compared to unstained media, were used to develop the compensation matrix and distinguish background staining from specific staining using *FloJo* ^TM^ software version 10.0 (Additional file 1: Figure S1B). Approximately 5% of the MVs stained positive for lactadherin, which binds phosphotidyl-serine (PS). Of the PS+ MVs, ethanol increased HMGB1 in a dose-dependent fashion up to 123% of controls at 75 mM (not shown).

### RIP assays

Media MVs were isolated by centrifugation as described above. The MV pellet was lysed in 0.1% Triton X-100 containing buffer. MV HMGB1 was immunopurified using Dynabeads® M-270 Epoxy (ThermoFisher 14321D) according to the manufacturer’s instructions as described [37]. Briefly, Dynabeads were coupled overnight to either an anti-HMGB1 antibody (Abcam ab18256) or anti-Ago2 (ab32381). MV protein was incubated with anti-HMGB1 coupled Dynabeads overnight at 4 °C. HMGB1 was then eluted from the Dynabeads and incubated in Trizol^TM^ buffer. Micro-RNA isolation was performed as above, with the same amount of total mRNA assessed for RT-PCR per sample. let-7b, miR-155, and miR181c were assessed as described above.

### Western blot

Human brain tissue was homogenized and sonicated in Tris lysis buffer containing 7.4% EDTA, 3.8% EGTA, and 1% Triton X-100. Lystates were centrifuged at 21,000*g* to remove the nuclear fraction. Samples were diluted in equal amounts of RIPA and DTT containing reducing buffer (Pierce TM catalog number 39000) to a final amount of either 30 or 40 μg protein per well. Samples were run on 4–15% Ready Gel Tris-HCL gel (BioRad) and transferred onto PVDF membranes (BioRad). Membranes were incubated overnight at 4 °C with primary antibody. Secondary incubation was performed the following day and membranes visualized and bands quantified using the LiCor Odyssey imaging system^TM^. Values for proteins of interest were normalized to beta actin expression for each subject.

### Assessment of neuronal cell death

The uptake of the fluorescent exclusion dye propidium iodide (PI) was used for determination of neuronal cell death. PI is a polar compound that is impermeable to a cell with an intact cell membrane but penetrates damaged cell membranes. Inside the cells, it binds nuclear DNA to generate the brightly red fluorescence. This method has been well characterized as accurately measuring neuronal degeneration in organotypic slice cultures [38]. For each experiment, PI was added into the culture medium at the beginning of treatment at a concentration of 5 μg/ml and PI fluorescence images were captured at indicated time points. PI fluorescent intensity was measured and analyzed with the AxioVision 3.1 software. Mean fluorescent density was quantified using ImageJ^TM^ software.

### Statistical analyses

Data are expressed as a mean values ± standard error of mean from the indicated number of slices or experiments. Student’s *t* tests were performed for two-group analyses. For concentration-response curves, a one-way ANOVA followed by Dunnett’s multiple comparisons test was utilized. Differences were considered to be statistically significant if *p* value of <0.05. For human brain tissue analyses, paired *t* tests were performed between alcoholic subjects and their matched controls. Pearson’s correlation test was performed to assess for correlations of normally distributed data.

## Results

### Postmortem human alcoholic brains have increased TLR7, HMGB1, and microglial activation

**Fig. 1:**
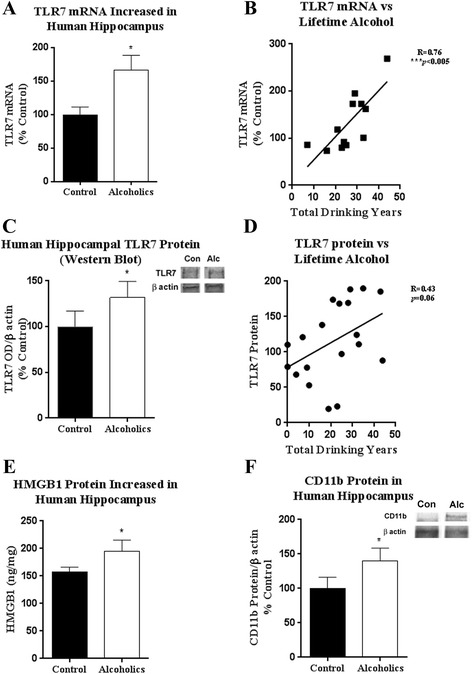
Postmortem human alcoholic hippocampus shows increased TLR7, HMGB1, and CD11b expression. Frozen hippocampal tissue from human alcoholics was obtained from the New South Wales Brain Tissue Bank. Either mRNA or protein was isolated. **a** TLR7 mRNA was 1.66-fold greater in alcoholics. **b** TLR7 mRNA correlated positively with total drinking years across all subjects (*R* = 0.76, ***p < 0.005). **c** TLR7 protein was measured by western blot. TLR7 was increased in the hippocampus of alcoholics by 31%. **d** TLR7 protein was also related to lifetime alcohol consumption assessed by total drinking years. Pearson’s coefficient *R* = 0.43, *p* = 0.06. **e** HMGB1 protein was increased in the hippocampus of alcoholics by 24% (195.5 ± 20.04 vs 157.8 ± 8.22 ng/mg total protein, alcoholics vs control, mean ± SEM). **f** CD11b protein expression was increased by 40% in alcoholics showing microglia activation. *Con*-healthy controls, *Alc*-alcoholics (ALC). *p < 0.05, paired *t* test, *N* = 8–10 subjects per group

Previous studies have found alcoholic hippocampal neurodegeneration [17], increased microglial markers and cytokine expression [39], and TLR7 induced neuroimmune activation and neurodegeneration [6]. TLR7 activation leads to neuroimmune activation as well as neurodegeneration [6]. However, a role for TLR7 in alcoholism has not been described. To assess the involvement of TLR7 and microglia in the pathology of alcoholism, we obtained frozen hippocampal tissue of postmortem human alcoholics from the New South Wales (NSW) Brain Tissue Bank. Demographics and alcohol use history of alcoholics and healthy control subjects are shown in Table 2. We found no significant differences between groups in age (*p* = 0.98), PMI (*p* = 0.73), brain pH (*p* = 0.53), or RIN values (*p =* 0.9). RIN values averaged 7.28 and 7.2 in control and alcoholic groups, respectively, demonstrating similar and adequate mRNA integrities for RT-PCR. Further, amplification occurred prior to 40 cycles and single peak MELT curves were seen consistent with detection of the target mRNA of interest. Hippocampal tissue from alcoholics showed increased expression of TLR7 mRNA (166%, *p* < 0.05) than control moderate drinkers (Fig. 1a). TLR7 mRNA correlated positively with lifetime alcohol consumption as assessed by total drinking years (Fig. 1b, *R* = 0.76, *p* < 0.005) and kg alcohol/lifetime (*R* = 0.76, *p* < 0.005, not shown) across all subjects. TLR7 protein was also increased in postmortem hippocampal tissue from human alcoholics (Fig. 1c). TLR7 protein showed a trend toward a positive correlation with total drinking years (Fig. 1d) that approached significance (Pearsons coefficient *R* = 0.43, *p* = 0.06). Neuroimmune responses to TLR7 in vitro require HMGB1 [25]*.* In the hippocampus of alcoholics, HMGB1 protein was increased by 24% as measured by ELISA (Fig. 1e). To assess microglia, CD11b was measured by western blot. CD11b was increased in alcoholics by 40, consistent with microglial activation (Fig. 1f). Thus, the neuropathology of human alcoholism involves increased expression of TLR7, HMGB1, and microglial CD11b.

**Table 2:** Demographics of alcoholics and control subjects from New South Wales Brain Tissue Bank

DSM V alcohol classification	Age	PMI	Brain pH	RIN	Lifetime alcohol (kg)	Cause of death	Agonal state/mode of death
Control	24	43	6.27	6.2	15	Arrhythmia	Rapid
Control	40	27	6.79	7.4	47	Pulmonary embolus	Intermediate
Control	44	50	6.6	7.1	28	IHD	Rapid
Control	46	29	6.12	4.4	115	MI	Intermediate
Control	48	24	6.73	6.9	17	IHD	Rapid
Control	50	30	6.37	7.5	0	IHD	Rapid
Control	50	40	6.87	8.6	18	Hemopericardium	Rapid
Control	53	16	6.84	7.9	102	Cardiomyopathy	Rapid
Control	60	28	6.8	8	0	IHD	Rapid
Control	62	46	6.95	8.8	5	IHD	Rapid
Mean ± SEM	48 ± 3	33 ± 3	6.56 ± 0.1	7.28 ± 1	35 ± 13		
AUD, mild	25	43.5	6.7	6.9	552	CO and EtOH	Intermediate
AUD, moderate	42	41	6.5	8	1472	Bromoxynil/EtOH	Intermediate
AUD, remission	44	15	6.48	7.9	639	IHD	Rapid
AUD, severe	45	18.5	6.57	7.9	1799	Drowning	Intermediate
AUD, severe	49	44	6.41	6.4	1012	IHD	Rapid
AUD, severe	49	16	6.19	6.2	613	MI	Rapid
AUD, moderate	50	17	6.3	7.0	2453	IHD	Rapid
AUD, severe	50	34.5	6.93	7.3	5212	Acute bronchitis	Intermediate
AUD, severe	61	59	6.57	6.1	8052	Myocarditis	Intermediate
AUD, severe	61	23.5	6.92	8.3	5621	IHD	Rapid
Mean ± SEM	48 ± 3	31 ± 5	6.63 ± 0.1	7.2 ± 1	2743 ± 829		

Detailed clinical data was collected for each subject as described in the “Methods” section. All subjects were male. *AUD* alcohol use disorder, *PMI* postmortem interval, *IHD* ischemic heart disease, *MI* myocardial infarction, *CO* carbon monoxide. Agonal state terminal phase durations—rapid: <1 h, intermediate: 1–24 h, long term >24 h

### Ethanol increases TLR7 expression and its ligand let-7b and causes neuroimmune gene induction in rat HEC slice culture

**Fig. 2:**
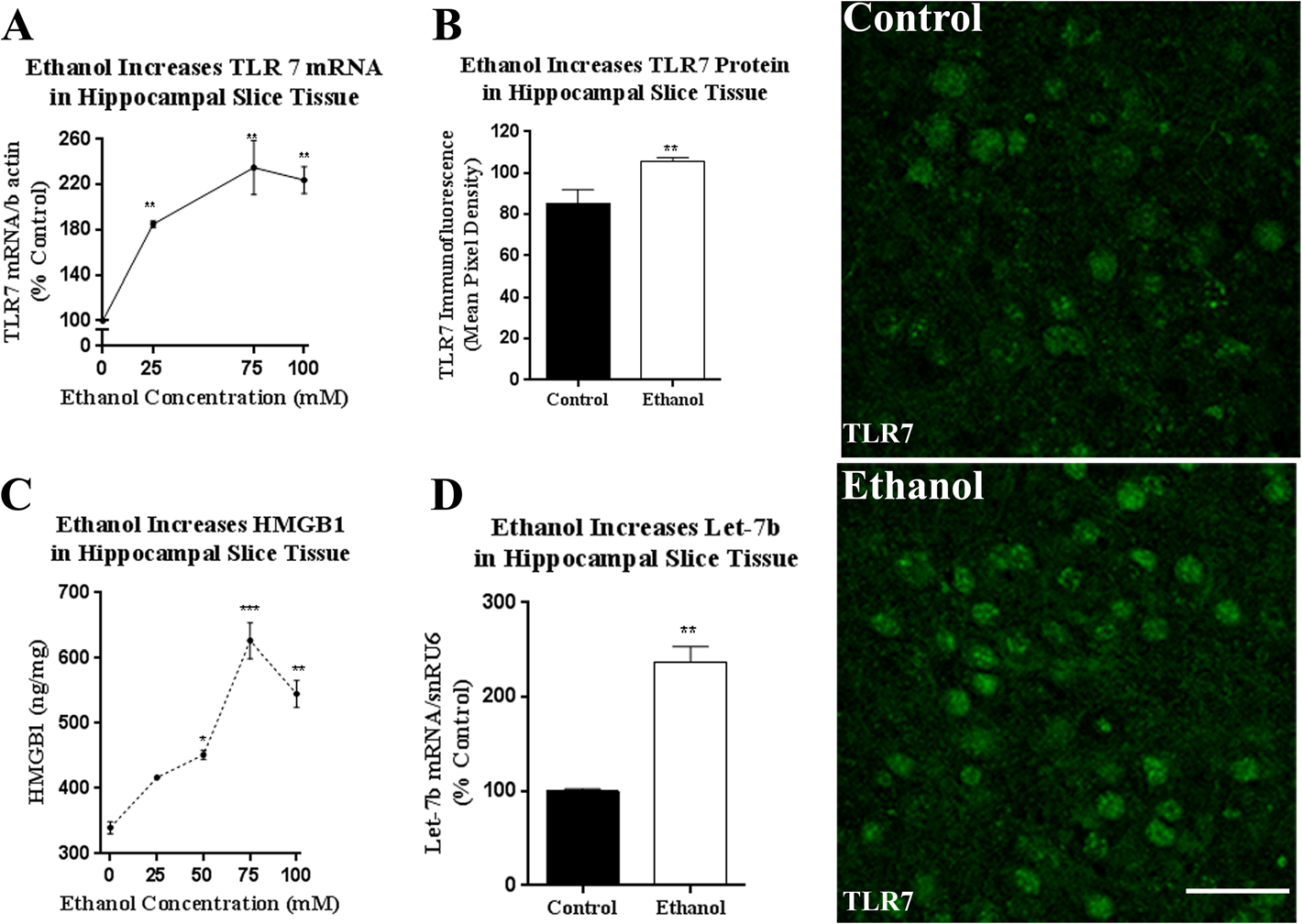
Ethanol increases miR let-7b, HMGB1 in hippocampal-entorhinal slice culture (HEC). HEC slices were exposed to ethanol for 48 h. Tissue was collected for miRNA, mRNA, protein, or immunohistochemistry. Levels of miR let-7b, IL-1β, and TNFα mRNA were measured in brain slice tissue using RT-PCR. TLR7 immunoreactivity (TLR7 + IR) was performed and mean pixel density measured. **a** TLR7 mRNA expression was increased by ethanol in a dose dependent fashion, up to twofold control levels. **b** A 23% increase in TLR7 + IR (protein) was found (105.5 ± 1.9 vs 85.3 ± 6.7 mean pixel density ± SEM, Ethanol vs Control, **p = 0.01, *t* test, *N* = 5–6 per group). **c** HEC was exposed to ethanol (25–100 mM) for 48 h. Ethanol increased HMGB1 protein (by ELISA) in the slice tissue by up to 85% in a concentration-dependent fashion. (One-way ANOVA *F*(4,5) = 48.07, *p* < 0.0005, Dunnett’s multiple comparisons post-test *p < 0.02,***p < 0.001). **d** miR let-7 was increased 2.4-fold at 48 h of ethanol exposure (237 ± 16.0% vs 100 ± 2.4%, Ethanol vs Control, mean ± SEM, **p < 0.01, *t* test

In order to investigate the role of ethanol induction of TLR7 on signaling, we utilized the hippocampal-entorhinal (HEC) slice culture. The miR let-7 is an endogenous ligand for TLR7 that results in neurodegeneration [6] and let-7 isoforms are increased in the brains of human alcoholics [9]. Thus, we hypothesized that ethanol would increase TLR7 and let-7 expression in vitro contributing to neuroimmune activation. The let-7b isoform in particular activates TLR7. Ethanol increased let-7b expression in HEC slice tissue by more than twofold at 48 h of exposure (Fig. 2a). Other let-7 family members in addition to other pro-inflammatory miRNAs were assessed and are shown in Additional file 4: Table S1. This was associated with an increase in TLR7 mRNA and protein (Fig. 2b, c). Similar to postmortem human tissue, ethanol increased HMGB1 protein levels in HEC slice culture in a concentration dependent fashion (Fig. 2d). Significant increases in HMGB1 were seen at 50 mM ethanol (33%, **p < 0.01), with a trend toward an increase at 25 mM (*p =* 0.056). Thus, ethanol treatment of HEC brain slice cultures increases expression of TLR7, let-7b, and HMGB1.

**Fig. 3:**
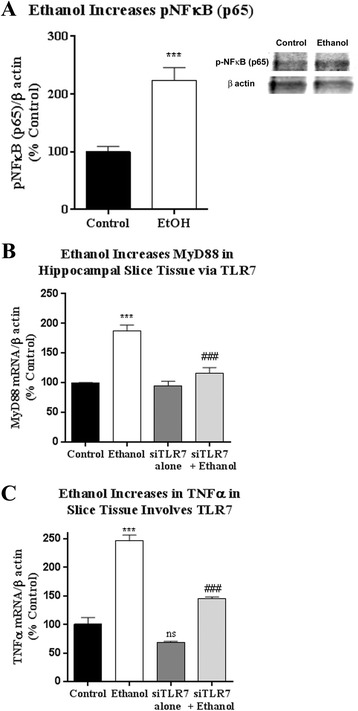
Induction of NFκB target genes by ethanol involves TLR7. HEC slices were exposed to ethanol for 48 h. mRNA was isolated from the tissue as described in the “Methods” section. Additional groups were incubated with siRNA against TLR7 (siTLR7) to assess for involvement of TLR7 role in induction of NFκB genes. **a** Ethanol caused phosphorylation of the NFκB-p65, evidenced by a 2.2-fold increase in pNFκB (p65) relative to controls (***p < 0.0003, *t* test). Insert shows representative western blot samples of pNFκB-p65 and β-actin. **b** MyD88 mRNA was increased by ethanol by 87% of control. siRNA against TLR7 returned MyD88 to near control levels. **c** TNFα mRNA was induced by ethanol by 246% at 48 h of ethanol exposure; 246.4 ± 10.243% vs 100.7 ± 11.8%, Ethanol vs Control, mean ± SEM. siRNA against TLR7 significantly reduced TNFα mRNA levels to 146% of control. *Error bars* denote mean ± SEM, ***p < 0.001 vs control, *###p* < 0.001 vs ethanol. *N* = 2–10 slices per group

We next investigated the role of TLR7 in ethanol-induced innate immune gene induction. TLR signaling involves MyD88 coupling to other signaling proteins leading to NFκB transcription of proinflammatory cytokines [7]. Indeed, we found that ethanol increased phosphorylation of the p65 NFκB (pNFκB-p65) by nearly 2.2-fold (Fig. 3a, 223.8 ± 22.15% vs 100 ± 9.2%, ethanol vs control, ***p < 0.0003 *t* test). NFκB-associated innate immune gene induction by ethanol was further evidenced by increased levels of MyD88, a key TLR signaling protein (187% increase, Fig. 3b) and TNFα mRNA (249% increase, Fig. 3c), and IL-1β (954% increase, not shown). The TLR7 agonist imiquimod also caused increases in TNFα and IL-1β (Additional file 2: Figure S2). We used an siRNA against TLR7 (siTLR7) to assess the effect of TLR7 signaling after ethanol. TLR7 siRNA effectively reduced TLR7 mRNA levels by 50% (100% ± 12.25 vs 49.6 ± 3.16, *p* < 0.0001; mean ± SEM; siControl vs siTLR7). MyD88 and TNFα gene expression caused by ethanol were reduced by 81 and 69% respectively (Fig. 3b, c). TLR7 can also activate IRF7 leading to interferon (IFN) induction. We observed a slight increase in IFNα mRNA (29% above control, *p <* 0.05, not shown) and a decrease IFNγ mRNA (52% reduction, *p <* 0.05, not shown) in response to ethanol. Thus, ethanol concomitantly induces activation of both TLR7 and its endogenous ligand, let-7b. Ethanol causes NFκB activation with associated induction of MyD88, a key TLR adapter protein, as well as proinflammatory cytokines associated with TLR7 activation.

### Ethanol releases miR let-7b and HMGB1 complexes in MVs from microglia

**Fig. 4:**
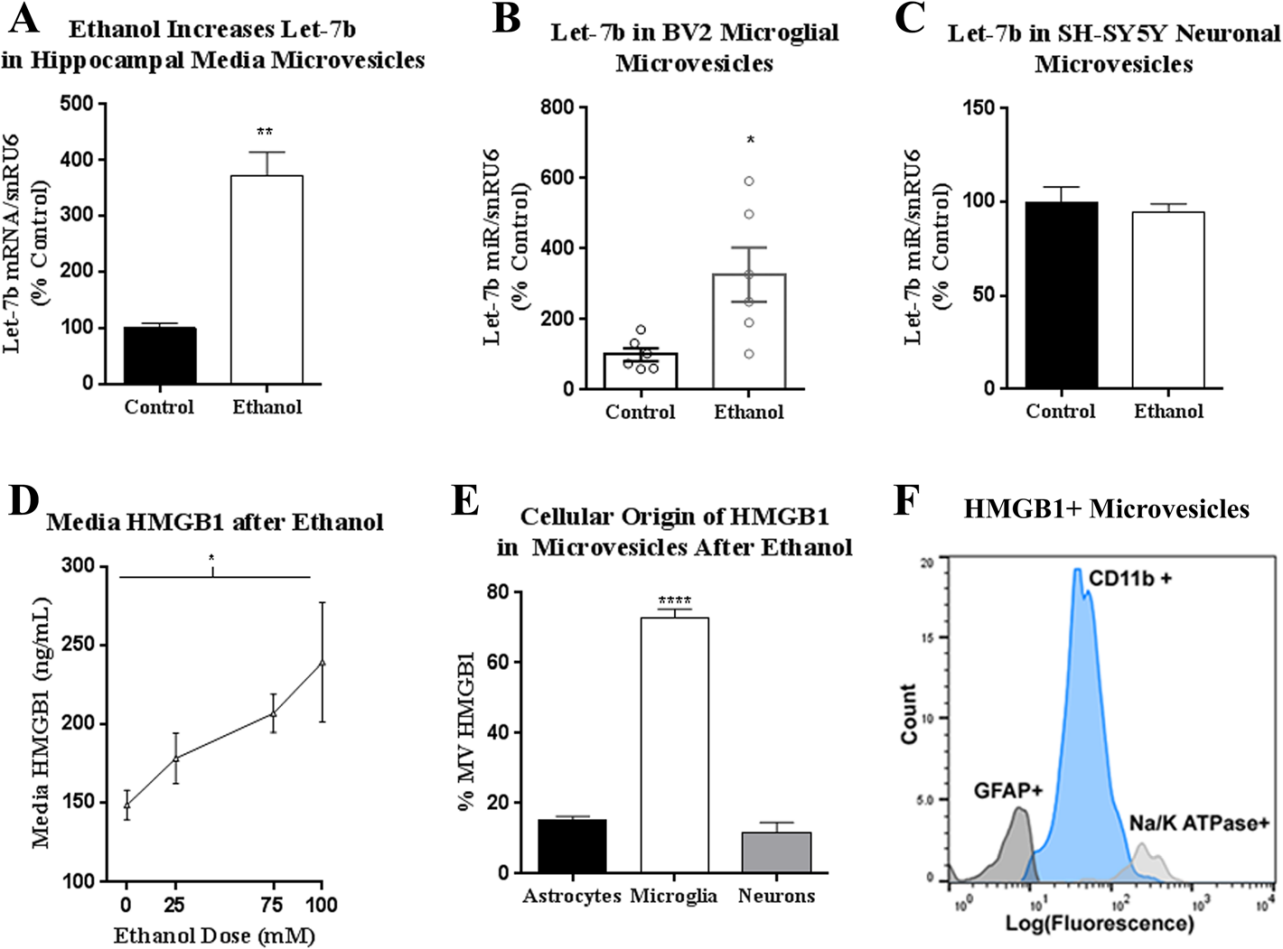
Ethanol causes microvesicle (MV) release of let-7b and HMGB1 from microglia. **a** MVs were isolated from hippocampal-entorrhinal slice culture (HEC) media after ethanol exposure. let-7b was increased in MVs 3.7-fold by ethanol. **b** Ethanol increased let-7b in BV2 microglia-derived MVs by 3-fold; 326.7 ± 76.3% vs 100 ± 18.2%, Ethanol vs Control, mean ± SEM, *p < 0.05, *t-*test. **c** SH-SY5Y neurons were treated with 100 mM ethanol for 24 h. Ethanol had no effect on let-7b release in MVs from SH-SY5Y neurons; 95.75 ± 5.2% vs 100 ± 8.1%, Control vs Ethanol, mean ± SEM. **d** Ethanol increased HMGB1 secretion into HEC culture media in a dose dependent fashion. **e** 72.9% of HMGB1+ MVs from HEC media are microglial-derived, whereas astrocytes and neurons comprised 15.3 and 11.8%, respectively. One-way ANOVA with Sidak’s multiple comparisons test, ****p < 0.00001. **f** Representative histogram of one ethanol treated sample showing populations of astrocytic (GFAP, *dark gray*), microglial (CD11b, *light blue*), and neuron-derived HMGB1+ MVs (Na/K ATPase α3, *light gray*). *p < 0.05, **p < 0.01, mean ± SEM, *N* = 3–6 per group

let-7b can be released from cells in extracellular microvesicles (MVs) to exert its effects on recipient cells [40, 41]. HMGB1 is also released in vesicle response to inflammation or cellular stress [29, 42]. Therefore, we assessed whether let-7b and HMGB1 were released in MVs in response to ethanol, as this could serve as an inter-cellular communication signal. We isolated vesicles based on their size (0.1–1 μm). This population is heterogeneous and may include both membrane-derived microparticles and larger exosomes. Ethanol caused a nearly fourfold increase in release of let-7b in media MVs (Fig. 4a). We then asked which cell type was responsible for microvesicular let-7 and HMGB1 release. We utilized specific cell lines to determine whether neurons or microglia release let-7b in MVs. Ethanol caused a threefold increase in MV let-7b from BV2 microglia (Fig. 4b) but not SH-SY5Y neurons (Fig. 4c), suggesting microglia are the source of secreted let-7. Ethanol also caused a dose-dependent increase in media HMGB1 (Fig. 4d). Thus, ethanol releases let-7b and HMGB1 from HEC brain slice cultures and BV2 microglia.

Consistent with previous findings, ethanol stimulated the release of HMGB1 in the absence of detectable cell death as assessed by propidium iodine uptake (Fig. 7b, c), consistent with active release from cells, rather than passive release from necrotic cells [22]. Therefore, we assessed ethanol-treated HEC slice media MVs to determine which cell types were secreting HMGB1. Using flow cytometry of HEC media, we were able to identify the cellular origin of HMGB1+ MVs. Media MVs were labeled with fluorescent antibodies to HMGB1, GFAP (astrocytes), CD11b (microglia), and Na^+^/K^+^ ATPase α3 (neurons). Flow cytometric analysis revealed that 73% of the HMGB1-positive MVs were CD11b positive, indicating microglial origin. Approximately 15.3 and 11.8% were positive for GFAP and Na^+^/K^+^ ATPase α3 indicating astroglial and neuronal origins, respectively (Fig. 4e, f). Thus, ethanol causes secretion of HMGB1 in MVs primarily from microglia, as well as increasing let7b secretion in MVs.

**Fig. 5:**
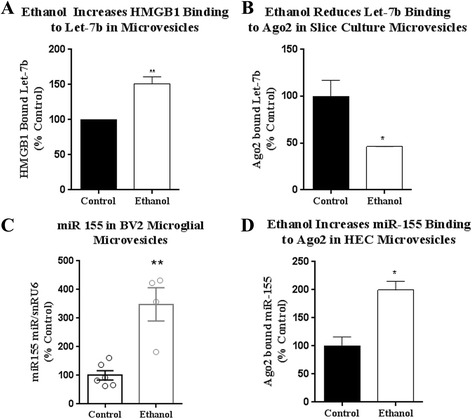
Ethanol alterations in miRNA expression and chaperone protein binding in microvesicles (MVs). Hippocampal-entorhinal (HEC) slices were treated with ethanol (100 mM) and RNA immunopurification (RIP) assay was utilized to assess let-7b complex formation with chaperone proteins. **a** RIP assay found that ethanol increases the association of let-7b with HMGB1 in MVs by 50%. **b** Ethanol reduces let-7b binding to argonaut (Ago2) by 50%. **c** Ethanol caused a robust increase in miR-155 in BV2 microglial MVs. **d** miR-155 binding to Ago2 was increased in HEC media MVs after ethanol treatment. Mean ± SEM, *p < 0.05, *N* = 3–6 per group

In addition to its DAMP actions as a TLR4 agonist, HMGB1 also acts as a chaperone for nucleic acids, steroid hormones [28], or cytokines [26] facilitating their binding to their own receptors. Given the “chaperone-like” nature of HMGB1 for nucleic acids, and our finding of let-7b and HMGB1 in MVs derived from microglia, we hypothesized that HMGB1 binds miR let-7b in MVs. Micro-RNAs normally bind argonaute 2 (Ago2) during the formation of the mature RNA-induced silencing complex (RISC) [43]. However, miRNAs targeted for secretion in MVs, rather than for intracellular regulation of mRNA stability might involve different chaperone proteins. In the case of let-7b, less than 20% of let-7b is bound to Ago2 in MVs from human plasma [44]. Therefore, we assessed let-7b binding to Ago2 and HMGB1 in MVs. Using an RNA immunopurification (RIP) assay, HMGB1 was immunoprecipated from HEC media MV followed by RT-PCR for miR let-7b. Ethanol was found to increase the association of let-7b with HMGB1 in MVs by 50% (Fig. 5a). Concomitantly, ethanol reduced the association of let-7b with its classical chaperone protein argonaute (Ago2) [37] in microvesicles by approximately 50% (Fig. 5b). In order to determine if this ethanol effect showed specificity for let-7b, we assessed two additional relevant pro-inflammatory miRNAs, miR-155 and miR181c. The miR-155 has been shown previously to be increased by 2.5-fold in plasma MVs in response to ethanol [24]. HMGB1 binding to miR181c was non-detectable after ethanol treatment (not shown). We found that ethanol increases miR-155 levels by 6.7-fold in our MV preparations (100 nm–1 μm) from BV2 microglia (Fig. 5c). Ethanol did not, however, increase the binding of HMGB1 with miR-155 (not shown), though miR-155 binding to Ago2 binding was increased as expected (Fig. 5f). Thus, ethanol is altering miRNA-chaperone binding in MVs. Ethanol-induced increases in let-7b binding to HMGB1 appear to be unique from other ethanol-induced miRNAs. This could be associated with the targeting of miRNA to MVs for secretion rather than to the RISC complex for regulation of mRNA stability and should be investigated in future studies. Regardless, ethanol increased HMGB1-let-7b complexes in MVs released from HEC brain slices.

### Ethanol enhances TLR7 mediated neurodegeneration, a requirement of HMGB1

**Fig. 6:**
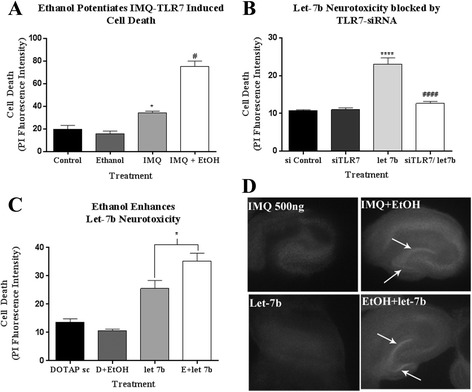
Ethanol enhances TLR7-mediated neurotoxicity. Hippocampal-entorhinal (HEC) slices were treated with TLR7 agonist Imiquimod (IMQ, 500 ng/mL), let-7b (5 μM), or ethanol (100 mM) for 48 h. Cell death was assessed by fluorescent propridium iodide (PI) uptake. **a** Brain slice cultures were treated with a low dose of IMQ (500 ng/mL) or vehicle for 48 h, followed by addition of either ethanol (100 mM) or vehicle for 4 days to assess whether TLR7 pre-sensitization with IMQ would result in ethanol induced neurotoxicity. IMQ treatment caused a 75% increase in PI staining over control. Ethanol robustly increased neurotoxicity (284% greater than control and 119% greater than IMQ alone) *p < 0.05 vs control, #*p <* 0.05 vs IMQ alone, *N* = 7–14 slices per group. **b** let-7b caused neurotoxicity in HEC slice tissue as shown by a greater than twofold increase in PI uptake relative to control. siRNA against TLR7 mRNA (siTLR7) prevented let-7 induced neurotoxicity. *p < 0.05 vs control, #*p <* 0.05 vs let-7b alone, *N* = 7–14 slices per group. **c** Ethanol potentiated let-7b-induced neurotoxicity (37.05 ± 2.4 vs 23.1 ± 1.7 mean fluorescent intensity) ****p < 0.0001 vs siControl, *####p* < 0.0001 vs let7b, *t* test, *N* = 10 slices. **d** Representative images of PI staining in hippocampal region of HEC slices. *Arrows* show neurotoxicity in dentate gyrus and CA1. Ethanol enhanced IMQ-TLR7 and let-7b induced neurotoxicity

Since ethanol increases TLR7 expression, let-7 release, and HMGB1 release, we hypothesized that the ethanol-induced increase in TLR7 expression primes TLR7 signaling and increases vulnerability to neurotoxicity due to TLR7 activation. To model this, while preventing other potential confounding effects of ethanol treatment, we used a low dose of the TLR7 agonist imiquimod (IMQ) to specifically prime TLR7 signaling, then assessed the effects of ethanol. Indeed, a low dose of IMQ resulted in a moderate increase in neurodegeneration, with ethanol addition after TLR7 priming causing nearly a fourfold increase in neurodegeneration (Fig. 6a). Next, we investigated the effect of ethanol on the endogenous TLR7 agonist, let-7b. First, we confirmed that let-7b causes neurodegeneration through TLR7 in our culture using siRNA to TLR7 and a let-7b mimic (Fig. 6b). We then found that ethanol does indeed potentiate let-7b-induced neurotoxicity (Fig. 6c). Ethanol also potentiated neuroimmune responses to a low dose of non-neurotoxic let-7b mimic (100 nM), increasing IL-1β expression by fourfold greater than let-7b alone (not shown). Thus, sensitization of TLR7 signaling with either IMQ or let-7 results in increased neurotoxicity to ethanol.

**Fig. 7:**
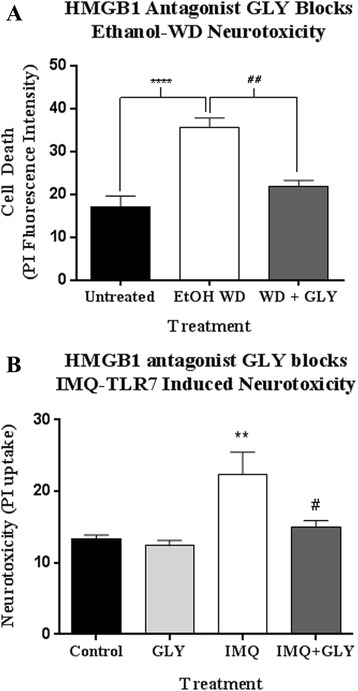
Ethanol and TLR7-induced neurodegeneration require HMGB1 release. **a** Hippocampal brain slice cultures were treated with ethanol or vehicle alone for 48 h followed by 24 h of ethanol withdrawal (WD) to induce neurotoxicity +/− HMGB1 antagonist glycyrrhizin (GLY, 100 μM). Ethanol resulted in neurotoxicity during withdrawal as assessed by propridium iodide uptake (35.5 ± 2.32 vs17.08 ± 2.5, mean ± SEM) ****p < 0.0001 vs control. GLY prevented neurotoxicity during withdrawal. *##p* < 0.01. **b** Hippocampal brain slice cultures were treated with the TLR7 agonist imiquimod (IMQ, 10 μg/mL) or vehicle for 48 h +/− GLY (100 μM). Neurotoxicity was assessed by PI uptake. IMQ treatment caused neurodegeneration with a 67.6% increase in PI uptake. This was diminished greatly by GLY (97.4% reduction). **p < 0.01 vs control, *#p* < 0.05 vs IMQ alone. *N* = 7–10 slices per group

Our finding of increased HMGB1 binding to let-7b in MVs after ethanol, coupled with previous observations of HMGB1 involvement in TLR7 agonist activation [25], suggested that HMGB1 may be acting as a chaperone, facilitating let-7b secretion in MV and binding to TLR7. In the HEC slice culture model, ethanol treatment alone causes neurotoxicity during withdrawal, i.e., following ethanol removal and involves glutamate excitotoxicity [45–49]. However, we have shown previously that glutamate toxicity requires HMGB1 release [50]. Thus, we hypothesized that HMGB1 release would be involved in ethanol withdrawal-induced neurotoxicity. Indeed, we observed that HMGB1 is required for ethanol-induced neurotoxicity (Fig. 7a). The HMGB1 antagonist glycyrrhizin (GLY) blocks both HMGB1 binding and release [51, 52]. GLY protected against ethanol-induced neurotoxicity during withdrawal. Further, let-7b was increased in MVs by 28% during ethanol withdrawal (not shown). HMGB1 has been shown to be required for TLR7-dependent immune responses in vitro [25]. Thus, we also investigated if HMGB1 is required for TLR7-induced neurodegeneration. We found that HMGB1 inhibition with GLY reduced neurodegeneration caused by the TLR7 agonist IMQ, with protecting against TLR7 mediated cell death (Fig. 7b). Thus, HMGB1 release is critical role in for neurotoxicity due to both ethanol and TLR7 activation. Neurodegeneration in human alcoholism involves cycles of chronic binge ethanol exposures and withdrawals. Our findings indicate that alcohol causes microglia to release let-7 and HMGB1 in MVs while concomitantly increasing TLR7 expression, leading to neurodegeneration. Thus, ethanol increases TLR7-induced neurodegeneration, through induction of TLR7, let-7 release, and HMGB1 secretion.

## Discussion

**Fig. 8:**
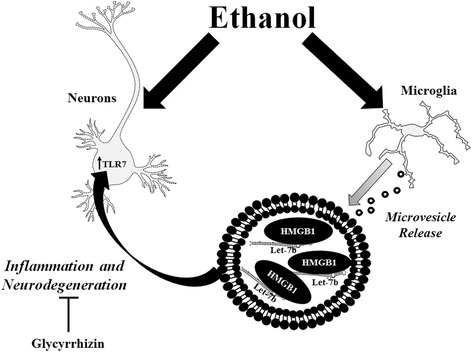
Proposed mechanism of HMGB1 and let-7 release in microvesicles. Ethanol causes an increase in TLR7 expression, and release of let-7b and HMGB1 from microglia in microvesicles, potentiating neurodegeneration. Ethanol increases the binding of let-7 with HMGB1 in microvesicles. HMGB1 inhibition with glycyrrhizin prevented TLR7 mediated neurotoxicity

The identification of endogenous ligands for TLRs has transformed the concept of inter-cellular signaling and innate immune activation. Let-7 release in MVs in particular has also been implicated in multiple inflammatory pathologies related to cancer, cardiovascular disease, and neurodegeneration [6, 53, 54]. We report here that ethanol exposure increases TLR7 expression and release of let-7b in microglia-derived microvesicles to promote neurodegeneration (Fig. 8). TLR7 mRNA and protein, as well as the microglial activation marker CD11b were increased in postmortem hippocampal tissue of alcoholics consistent with our in vitro findings. TLR7 mRNA changes were greater than that of TLR7 protein. This may be due to many factors such as translational repression [55]. The correlation of TLR7 expression with lifetime consumption of alcohol suggests a role of TLR7 signaling in the pathology of alcoholism. Ethanol also increased the binding of let-7b to the DAMP and chaperone HMGB1, while reducing let-7b binding to Ago2. This shifting of the chaperone protein association of let-7b might alter its function or initiate targeting to microvesicles. Traditional dogma is that miRNAs bind with Ago2 during the formation of the RISC complex and subsequent regulation of mRNA stability [43]. The export of miRNAs in MVs as potential TLR7 agonists is a new and emerging concept. Little is known about the targeting of miRNAs to vesicles for release versus to the cytosol for mRNA regulation. Future studies are warranted to better understand how different miRNA chaperone proteins, such as HMGB1 versus Ago2, differentially effect miRNA destination and function. HMGB1 is known to be secreted in microvesicles [42, 56] and might escort let-7 to microvesicles for secretion, rather than to the Ago2-associated RISC complex, where it would subsequently act intracellularly to regulate the stability target mRNAs. We also found that HMGB1 inhibition prevented TLR7-mediated neurotoxicity. This is consistent with a previous report showing that HMGB1 is required for TLR7-mediated immune responses in mouse embryonic fibroblasts [25]. Simultaneous activation of TLR4 and TLR7 with LPS and loxorobine respectively did not result in increased toxicity in mixed neuronal-microglia cultures [57]. Therefore, we think the requirement for HMGB1 involves facilitating the interaction of let-7 with TLR7, rather than a requirement for co-stimulation of TLR4 by HMGB1. The importance of miRNA signaling has been identified in several aspects of alcohol use disorders. During fetal alcohol exposure, ethanol disrupts miRNA profiles to modulate neurodegeneration and proliferation [58, 59]. In adults, ethanol causes systemic release of miR-155 and miR-27a to regulate TLR4 signaling and monocyte activation state, respectively [60, 61]. Many miRNAs, including let-7 isoforms, are upregulated in the brains of humans and mice after chronic alcohol [8, 9]. We identify a role of let-7 in the pathology of alcoholism that involves inter-cellular signaling through TLR7, rather than its intracellular function involving mRNA stabilization.

We also found that HMGB1 was required for ethanol withdrawal-induced neurotoxicity. Studying withdrawal in the HEC slice culture model is critical, since ethanol exposure itself does not cause cell death. Consistent with previous studies using hippocampal slice culture, ethanol-induced neurotoxicity was seen during the ethanol withdrawal phase [45–49]. This withdrawal toxicity is known to involve glutamate release [47]. However, we found that inhibition of HMGB1 prevented ethanol withdrawal neurotoxicity. We have found previously that glutamate toxicity in the absence of ethanol also involves HMGB1 release, with glycyrrhizin preventing death [50]. The exact role of HMGB1 in withdrawal toxicity is not clear. However, TLR4 responses have been shown to be inhibited during the neurotoxic withdrawal phase in HEC cultures [49]. We found, however, that TLR7 responses during ethanol withdrawal are enhanced and require HMGB1 (Additional file 3: Figure S3). This also suggests that the requirement for HMGB1 during ethanol withdrawal might not involve its actions at TLR4, but might rather involve TLR7. We also found that let-7b is released during withdrawal. Future studies should investigate the role of HMGB1, let-7, and TLR7 in ethanol withdrawal-induced toxicity. Human alcoholism involves frequent exposures of chronic alcohol followed by withdrawal. Our findings suggest that recurrent TLR7 activation by ethanol-induced microglial let-7 and HMGB1 release contributes to the progressive neurodegeneration associated with alcoholism. This sensitization of microglia to release pro-inflammatory MVs may be similar in Parkinson’s, Alzheimer’s, and other neurodegenerative diseases.

It is important to note that high ethanol concentrations (>75 mM) were used in HEC slice culture and in vitro cell line experiments. However, human alcoholics reach very high blood alcohol concentrations (BACs). A report of 117 alcoholics showed that >57% had BACs from 43 to 125 mM [62], with alcoholics remaining functional at higher BACs [63]. Further, the in vitro findings of increased HMGB1 and TLR7 are seen in postmortem human alcoholics and are of similar magnitude. Thus, the concentrations used in our in vitro studies appropriately model the human condition. Also, it is important to note that different cell lines might have different features. We employed in vitro models from a variety of sources including rat hippocampal slice culture, mouse BV2 microglia, and human SY-SY5Y neurons. The rat hippocampal slice culture has all brain cell types present in their native configurations making it the best in vitro system we have employed. The combination of these tools shows that microglia are the primary source of let-7b in MVs in response to ethanol. Though the use of multiple systems strengthens our conclusions, other neuronal or microglial cell lines might show different responses and should be investigated in future studies.

Our findings elucidate a novel mechanism of inter-cellular communication in neuroimmune pathology (Fig. 8). Flow cytometric and cell line studies identified microglia as the primary source of the MV-secreted HMGB1 and let-7b. These findings are consistent with our previous in vivo findings of activated microglia following ethanol [19, 64, 65], evidence of microglial sensitization in postmortem human alcoholic brain [39], and the in vitro observation that the microglia are required for neuronal death due to agonists to TLR2, 4, and 9 [57]. Our findings further emphasize the need to develop microglia-targeted therapies for neuroimmune diseases. Also, the observation that ethanol increases the formation of HMGB1-let-7b complexes, and that HMGB1 inhibition prevents TLR7 induced neurotoxicity, uncovers a new potential for HMGB1 inhibition in preventing alcohol-induced and other neuroimmune pathologies. The investigation of the mechanisms of MV packaging of contents and secretion might also produce additional therapeutic targets. Further, several let-7 family members and other miRNAs were altered by ethanol in MVs (Additional file 4: Table S1), warranting further future investigation. The method of MV isolation used in this study yields vesicles between 0.1 and 1.0 μm in diameter. The origin of these particles may include exocytosis of multivesicular bodies, budding of the plasma membrane, or autophagy-associated secretory vesicles from living cells [66–68]. Identification of the mechanisms underlying this MV secretion may produce novel pharmacological targets for alcoholism or other conditions involving neuroimmune activation. In summary, we identify a novel inter-cellular neuroimmune mechanism involved in the pathology of alcoholism that provides multiple potential therapeutic targets.

## Conclusions

We find increased TLR7 in alcoholic hippocampus and with ethanol treatment of slice cultures. Ethanol increases TLR7 activation and releases of HMGB1-miR-let-7 complexes in microglia-derived vesicles that cause neurotoxicity via TLR7 activation. TLR7 activation by alcohol in humans may contribute to the neuropathology of alcoholism.

## Additional files

Additional file 1: Figure S1. Flow cytometric assessment of microvesicles. Microvesicles (MVs) were isolated by size using centrifugation (21,000*g* for 1 h) and analyzed by flow cytometry. (A) Depiction of size gating for microvesicles (0.1 to 1.0 μm) using MegaMix^TM^ gating beads. (B) Specific staining for cell-type markers was determined by comparison with unstained controls. Depictions of unstained (gray) and single color controls for each primary antibody are shown. These were used for determination of compensation matrix and identification of specific populations (arrows). (TIF 49286 kb)
Additional file 2: Figure S2. TLR7 agonist imiquimod (IMQ) increases TLR7, let-7b, and neuroimmune markers in brain slice tissue similar to ethanol. (A) TLR7 mRNA expression was increased by 3.8-fold by IMQ treatment (10 μg/mL, 48 h). (B) IMQ increased expression of IL-1β (peak 60-fold) and TNFα mRNA (peak 12-fold) after 24 and 48 h of exposure. (C) Media HMGB1 was increased by IMQ (10 μg/mL) rapidly within 8 h (threefold) and then reached a fourfold total increase at 48 h. (D) let-7b miR was increased by twofold in brain slice culture after 24 h exposure to IMQ (***p* < 0.01). (E) Imiquimod increased let-7b release in microvesicles from 24–48 h in a fashion similar to ethanol. let-7b levels were threefold control at 24 h and fivefold control at 48 h. Error bars denote mean ± SEM. **p* < 0.05, ***p* < 0.01 (TIF 2159 kb)
Additional file 3: Figure S3. Ethanol withdrawal enhances TLR7 neurotoxicity by HMGB1 release. HEC slices were treated with ethanol (100 mM), imiquimod (IMQ, 10 μg/mL) vehicle for 48 h. During the 24-h withdrawal period, slices either had vehicle, IMQ, or IMQ + glycyrrhizin (100 μM). Cell death was quantified using propridium-iodine uptake. (A) IMQ treatment during withdrawal produced greater neurotoxicity than either ethanol withdrawal or IMQ alone. HMGB1 inhibition was with GLY prevented the ethanol induced enhancement. (B) Representative images of each treatment condition. *N* = 7–10 slices. **p* < 0.05 vs control. *#p* < 0.05 vs IMQ alone or WD + IMQ. (TIF 2299 kb)
Additional file 4: Table S1. Effect of ethanol on selected miRNAs in hippocampal entorhinal cortex (HEC) slice culture microvesicles (MVs) and tissue. HEC sections were treated with ethanol (100 mM) for 48 hours. MVs and tissue were analyzed for miRNA expression by RT-PCR and expressed as percent of control ± SEM. **p* < 0.05, ***p* < 0.01, *t* test (DOCX 16 kb)

## Abbreviations

Ago2: Argonaute
DAMPs: Damage-associated molecular pattern molecules
GLY: Glycyrrhizin
HEC: Hippocampal-entorhinal cortex
HMGB1: High mobility group box 1 protein
IMQ: Imiquimod
miR: Micro-RNA
MV: Microvesicle
NSW: New South Wales
RIP: RNA immunoprecipitation
ss: Single stranded
TLR: Toll-like receptor
WD: Withdrawal
