# Supplementary figures and images for: Microglial-derived miRNA let-7 and HMGB1 contribute to ethanol-induced neurotoxicity via TLR7
Source: J Neuroinflammation. 2017 Jan 25;14:22. doi: 10.1186/s12974-017-0799-4 (PMC5264311; doi:10.1186/s12974-017-0799-4)

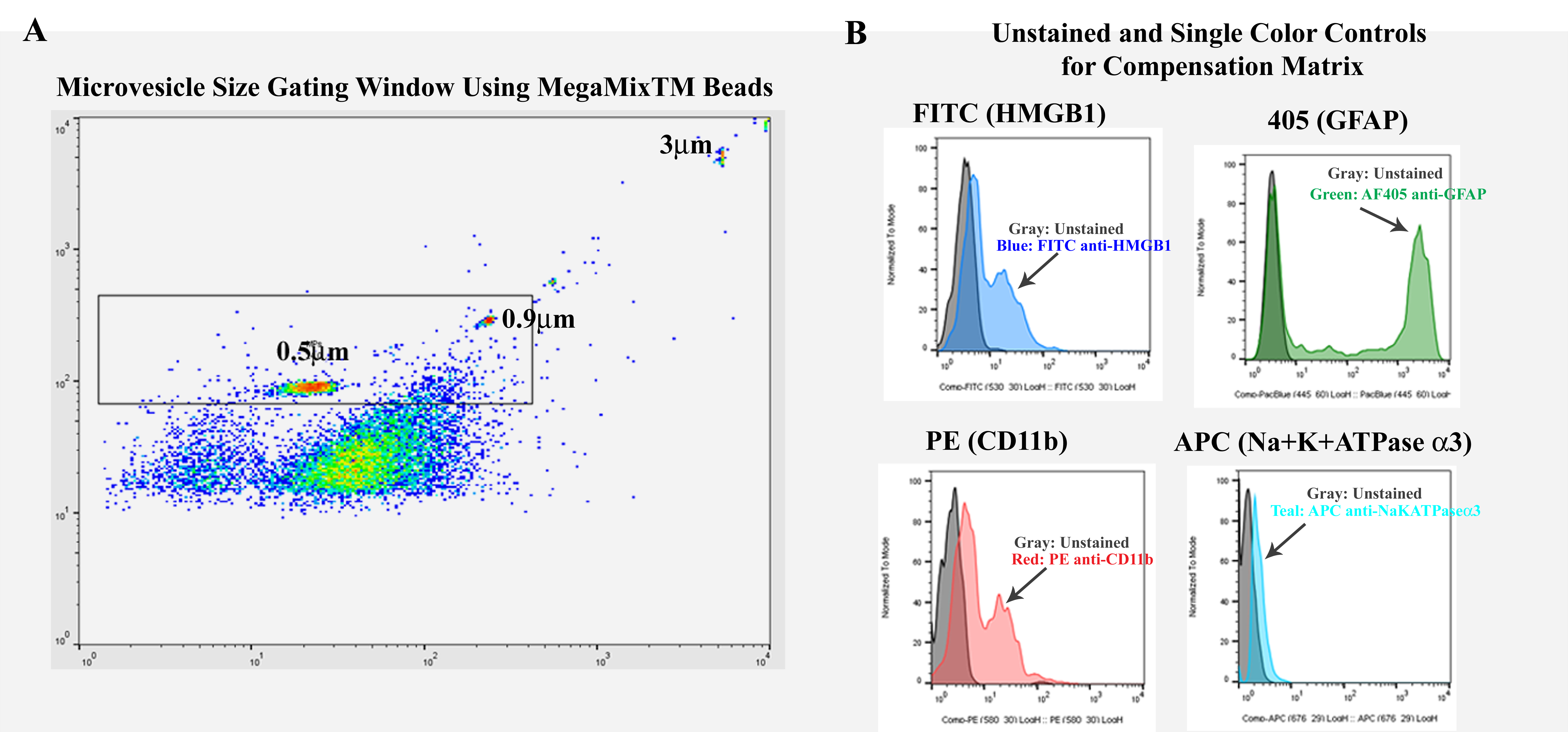

Supplement: Additional file 1: Figure S1. — Flow cytometric assessment of microvesicles. Microvesicles (MVs) were isolated by size using centrifugation (21,000g for 1 h) and analyzed by flow cytometry. (A) Depiction of size gating for microvesicles (0.1 to 1.0 μm) using MegaMixTM gating beads. (B) Specific staining for cell-type markers was determined by comparison with unstained controls. Depictions of unstained (gray) and single color controls for each primary antibody are shown. These were used for determination of compensation matrix and identification of specific populations (arrows). (TIF 49286 kb) [file 12974_2017_799_MOESM1_ESM.tif]

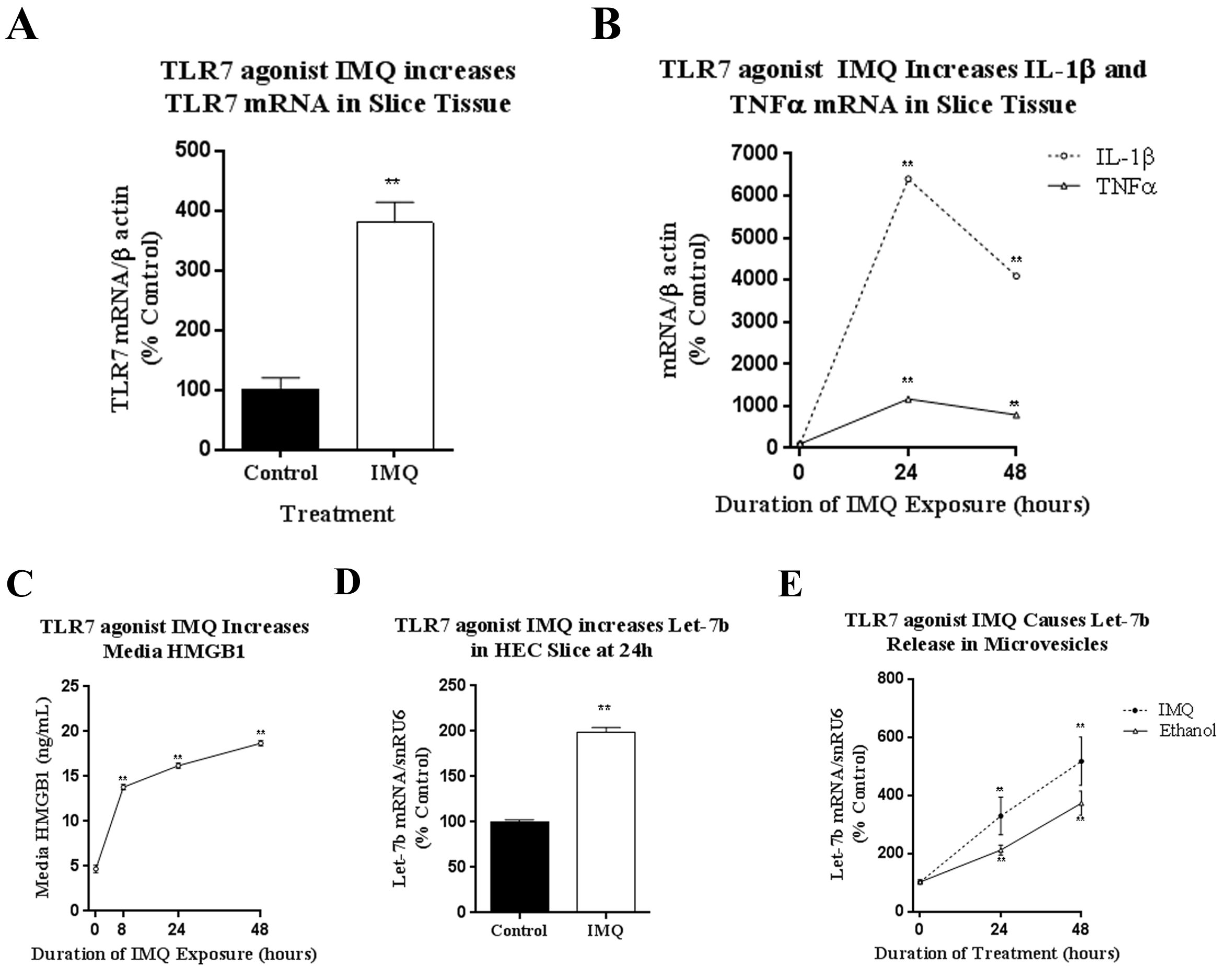

Supplement: Additional file 2: Figure S2. — TLR7 agonist imiquimod (IMQ) increases TLR7, let-7b, and neuroimmune markers in brain slice tissue similar to ethanol. (A) TLR7 mRNA expression was increased by 3.8-fold by IMQ treatment (10 μg/mL, 48 h). (B) IMQ increased expression of IL-1β (peak 60-fold) and TNFα mRNA (peak 12-fold) after 24 and 48 h of exposure. (C) Media HMGB1 was increased by IMQ (10 μg/mL) rapidly within 8 h (threefold) and then reached a fourfold total increase at 48 h. (D) let-7b miR was increased by twofold in brain slice culture after 24 h exposure to IMQ (**p < 0.01). (E) Imiquimod increased let-7b release in microvesicles from 24–48 h in a fashion similar to ethanol. let-7b levels were threefold control at 24 h and fivefold control at 48 h. Error bars denote mean ± SEM. *p < 0.05, **p < 0.01 (TIF 2159 kb) [file 12974_2017_799_MOESM2_ESM.tif]

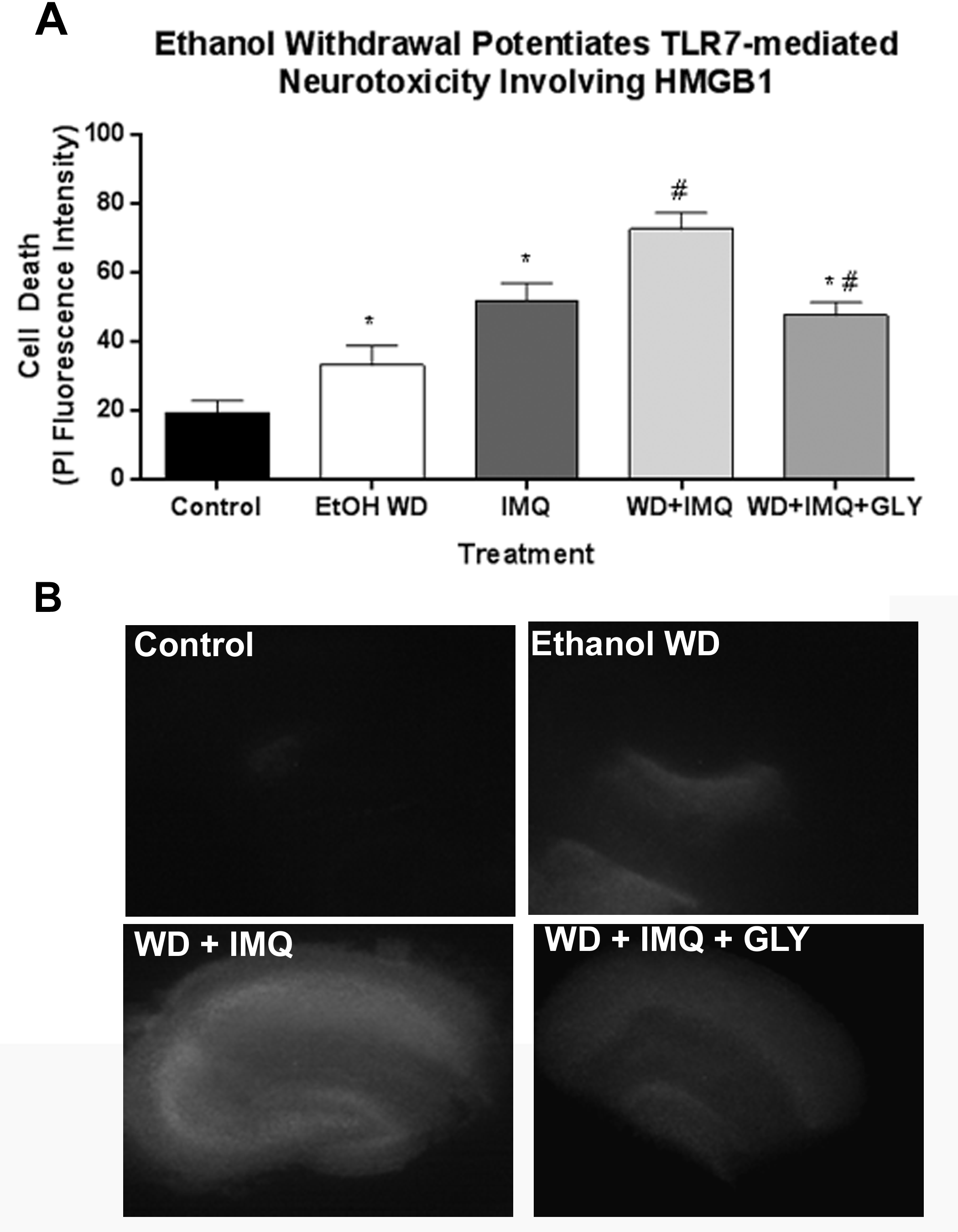

Supplement: Additional file 3: Figure S3. — Ethanol withdrawal enhances TLR7 neurotoxicity by HMGB1 release. HEC slices were treated with ethanol (100 mM), imiquimod (IMQ, 10 μg/mL) vehicle for 48 h. During the 24-h withdrawal period, slices either had vehicle, IMQ, or IMQ + glycyrrhizin (100 μM). Cell death was quantified using propridium-iodine uptake. (A) IMQ treatment during withdrawal produced greater neurotoxicity than either ethanol withdrawal or IMQ alone. HMGB1 inhibition was with GLY prevented the ethanol induced enhancement. (B) Representative images of each treatment condition. N = 7–10 slices. *p < 0.05 vs control. #p < 0.05 vs IMQ alone or WD + IMQ. (TIF 2299 kb) [file 12974_2017_799_MOESM3_ESM.tif]
