# Supplementary material for: Microglial-derived miRNA let-7 and HMGB1 contribute to ethanol-induced neurotoxicity via TLR7
Source: J Neuroinflammation. 2017 Jan 25;14:22. doi: 10.1186/s12974-017-0799-4 (PMC5264311; doi:10.1186/s12974-017-0799-4)
Supplement: Additional file 4: Table S1. — Effect of ethanol on selected miRNAs in hippocampal entorhinal cortex (HEC) slice culture microvesicles (MVs) and tissue. HEC sections were treated with ethanol (100 mM) for 48 hours. MVs and tissue were analyzed for miRNA expression by RT-PCR and expressed as percent of control ± SEM. *p < 0.05, **p < 0.01, t test (DOCX 16 kb) [file 12974_2017_799_MOESM4_ESM.docx]

| **Supplemental Table 1: Effect of ethanol on selected miRNAs in Hippocampal Entorhinal Cortex (HEC) Slice Culture Microvesicles (MVs) and Tissue.** HEC sections were treated with ethanol (100mM) for 48 hours. MVs and Tissue were analyzed for miRNA expression by RT-PCR and expressed as percent of control ± SEM. **p<0.05*, ***p<0.01, t-test* | | | | |
| --- | --- | --- | --- | --- |
| **miRNA** | **Media Microvesicles** | **Slice Tissue** | |  |
| Let-7a | 277 ± 25** | | 140 ± 6** | |
| Let-7b | 317 ± 9** | | 162 ± 11** | |
| Let-7c | 158 ± 6** | | 128 ± 6** | |
| Let-7d | 1176 ± 11** | | 135 ± 10** | |
| Let-7i | 160 ± 9** | | 149 ± 2** | |
| miR-21 | 210 ± 4** | | 175 ± 4** | |
| miR-155 | 334 ± 28** | | 172 ± 1** | |
| miR-181c | 174 ± 14** | | 272 ± 16** | |
